# American Academy of Dental Science, Annual Meeting

**Published:** 1900-04

**Authors:** 

**Affiliations:** American Academy of Dental Science


### AMERICAN ACADEMY OF DENTAL SCIENCE, ANNUAL MEETING.

The annual meeting and banquet of the American Academy of Dental Science was held at Young's Hotel, Boston, on Wednesday evening, November 15. About fifty members were in attendance.

There were present as guests of the evening, Harold Williams, M.D., Dean of the Medical and Dental Departments of Tufts College; Rev. Marcus D. Buell, D.D., Dean of the Theological School of Boston University; Rev. E. H. Byington, D.D., of Newton, Mass.; and Mr. John J. Enneking, of Boston.

Prior to the banquet, the regular business of the organization was transacted, and the following officers were elected for the ensuing year:

President, Dr. V. C. Pond; Vice-President, Dr. George F. Eames; Recording Secretary, Dr. Frank Perrin; Treasurer, Dr.

William Y. Allen; Corresponding Secretary, Dr. George H. Payne; Librarian, Dr. H. G. Hichborn; Editor, Dr. Charles H. Taft.

*Executive Committee.*—Drs. Frederick Bradley, F. G. Eddy, Thomas Fillebrown.

The post-prandial exercises were opened with a paper by Harold Williams, M.D., entitled "Education in Dental Medicine."

(For Dr. Williams's paper, see page 239.)

The Rev. Dr. Byington gave expression to the pleasure it gave him to be present, to the debt which he owed to the dental profession, and to the general thoroughness and fidelity with which dentists deal with their patients. The speaker pointed out some of the analogies between the work of dentists and that of preachers. He was of the opinion that the dentist should be an idealist, and that the most important and successful practical work in the world, of whatever nature, or connected with whatever profession, is done by those who are really idealists.

Among the thoughts that the Rev. Dr. Buell gave utterance to was this: That the most characteristic thing of every profession is *emergency;* that is to say, a situation suddenly confronting one, calling for the best thing that one can do, and giving no opportunity for special preparation. Emergencies make life fascinating, and the preparing of one's self to meet them is a work not only of to-day but of to-morrow,—a work that calls upon all our resources. The speaker was reminded of what happened when the French declared war against Germany: Von Moltke was sound asleep, when the door opened and he was informed that the French had declared war. He raised himself up on his arm and said, "Pull out the third drawer to the left there!" The drawer contained a complete plan of what was to be done in case war was declared. Von Moltke had studied war with France for something like forty years. He slept soundly the rest of the night, so the story goes.

"May I say, as a preacher may," said Dr. Buell, in conclusion, "God bless you in your great work. I must say, if I speak the truth, that your profession more and more is rising to the position where men of scientific training can inspect your work."

The next speaker, Mr. Enneking, outlined some of the essential conditions which must contribute to the making of good dentists and good artists. In either case, one must make sacrifices, and must so love his profession as to be willing to give up almost anything for it.

With the induction into office of the newly elected president, Dr. V. C. Pond, the meeting was brought to a close.

Charles H. Taft, D.M.D.,
*Editor American Academy of Dental Science.*

